# Native Lizards Living in Brazilian Cities: Effects of Developmental Environments on Thermal Sensitivity and Morpho-Functional Associations of Locomotion

**DOI:** 10.3389/fphys.2022.891545

**Published:** 2022-07-15

**Authors:** Nathalia Rossigalli-Costa, Tiana Kohlsdorf

**Affiliations:** Department of Biology, FFCLRP—University of São Paulo, Ribeirão Preto, São Paulo, Brazil

**Keywords:** development, eco-morphology, temperature, thermal physiology, Tropiduridae, urbanization

## Abstract

Environmental conditions often affect developmental processes and consequently influence the range of phenotypic variation expressed at population level. Expansion of urban sites poses new challenges for native species, as urbanization usually affects the intensity of solar exposure and shade availability, determining the thermal regimes organisms are exposed to. In this study, we evaluate the effects of different developmental conditions in a *Tropidurus* lizard commonly found in Brazilian urban sites. After incubating embryos of *Tropidurus catalanensis* in two different thermal regimes (Developmental Environments [DE]: cold 24°C and warm 30°C), we measured morphological traits in the neonates and quantified locomotor performance in horizontal and vertical surfaces at three temperatures [Test Temperatures (TT) = 24°C, 30°C and 36°C]. Results indicate effects of developmental temperatures on morphological features, expressing functional implications that might be decisive for the viability of *T. catalanensis* in urbanized areas. Lizards ran similarly on horizontal and vertical surfaces, and isolated analyses did not identify significant effects of DE or TT on the sprint speeds measured. Absolute Vmax (i.e., the maximum sprint speed reached among all TTs) positively correlated with body size (SVL), and neonates from the warm DE (30°C) were larger than those from the cold DE (24°C). Morpho-functional associations of absolute Vmax also involved pelvic girdle width and forelimb, hindlimb, trunk, and tail lengths. Emerging discussions aim to understand how animals cope with abrupt environmental shifts, a likely common challenge in urbanized sites. Our findings add a new dimension to the topic, providing evidence that temperature, an environmental parameter often affected by urbanization, influences the thermal sensitivity of locomotion and the morphological profile of *T. catalanensis* neonates. Thermal sensitivity of specific developmental processes may influence the ability of these lizards to remain in habitats that change fast, as those suffering rapid urbanization due to city growth.

## Introduction

Embryo development in oviparous vertebrates often involves exposure to environmental conditions that, even when buffered by the presence of protective membranes in the eggs or by parental care, may affect homeostasis and deviate from optimal conditions for growth and differentiation (Refsnider and Janzen, 2010; Noble et al., 2018). In lizards, for example, the choice of oviposition sites by females can affect the offspring, as incubation conditions influence traits such as hatching time, hatchling size, growth rate, and may ultimately determine the phenotypic profile of neonates (Warner and Andrews, 2002; Warner and Shine, 2008; Tiatragul et al., 2020). Among environmental parameters that usually affect development, temperature is probably one of the most relevant variables shaping the phenotype in ectotherm vertebrates (Angilletta, 2009). Thermal responses during ontogeny may produce different phenotypes (e.g., large or small neonate lizards; see Rossigalli-Costa et al., 2021) and can be addressed in the context of developmental plasticity and reaction norms (see Angilletta, 2009; Refsnider et al., 2019; Singh et al., 2020). When the thermal regimes change, species that exhibit behavioral flexibility or quickly respond to new conditions through plasticity in morphological or physiological traits tend to prosper in the novel environmental conditions (Winchell et al., 2016; Lapiedra et al., 2017; Noble et al., 2021).

Thermal relationships in embryos and tadpoles are receiving rising attention in the past decades (e.g., Clusella-Trullas and Chown, 2014; Bodensteiner et al., 2021; Hall and Sun, 2021; Taylor et al., 2021), a reflection of concerns from the scientific community with possible impacts of global warming on biodiversity (e.g., Moritz and Agudo, 2013; Diele-Viegas and Rocha, 2018; Diele-Viegas et al., 2020). The set of anthropic factors that accelerate climatic changes includes urbanization, a process that probably imposes the fastest and more radical changes in thermal regimes experienced by native species of ectotherm vertebrates. Expansion of urban centers involves the substitution of vegetated areas by impermeable surfaces, structural changes in habitat components (e.g., presence of walls and sidewalks), and modification of temperature and hydrological regimes due to changes in rates of sun exposure, shade availability and pollution (Forman, 2014). Urbanization may challenge some species to remain in their original distribution, as differences in environmental parameters between urban and natural sites might represent distinct selection pressures (Forman, 2014; Winchell et al., 2016), and lizards seem to be a particularly good system to evaluate phenotypic patterns and functional relationships with special attention to species found in urban sites (see Winchell et al., 2016; Johnson and Munshi-South, 2017). Studies focusing on urban lizards often interpret specific patterns in morphology (e.g., long limbs, more lamellae, larger toepads and wider bodies, see Winchell et al., 2016; Winchell et al., 2018b) and performance (e.g., high sprint speeds, see Winchell et al., 2018b) as adaptations to urbanization. Although most studies using urban lizards do not focus on embryo development, this is a relevant ontogenetic window because the choice of favorable microhabitats for oviposition in lizard species that lack parental care may buffer adverse conditions for embryo development imposed by specific environmental conditions (Shine and Harlow, 1996; Li et al., 2018), such as those derived from urbanization. On the other hand, in several species, the embryos may be very susceptible to environmental parameters (Wang et al., 2016; Bonnet et al., 2017) and pollutants (Lazić et al., 2015; Lazić et al., 2017) and, if the chosen oviposition site is not suitable, developmental plasticity may still enable proper growth and differentiation despite challenging environmental conditions (see Du et al., 2019). Plastic responses may comprise morphological and physiological traits as well as thermal sensitivity of specific functions. Shifts in incubation regimes may differentially affect specific sets of phenotypic traits (e.g., morphology, physiology, behavior; see Braña and Ji, 2000; Braña and Ji, 2007), and stability of functional associations—despite thermal sensitivity of particular developmental processes - might contribute for perpetuating the occurrence of some lizard species in urban sites.

The increasing number of studies addressing urbanization effects in different animals (see Rivkin et al., 2019) reflects an effort for understanding how new habitats (composed by buildings, impermeable surfaces at different orientations and high-density human population) might have functional consequences (e.g., Winchell et al., 2018a) that ultimately determine the phenotypic profile of populations of native species found in the cities. Some lineages, as the Neotropical *Tropidurus* lizards, are widely distributed and occupy different habitats, including species present in urban sites. The genus *Tropidurus* comprises habitat specialists but also generalist species (see Werneck et al., 2015), and records suggest that some species are relatively prone to occupy new niches and persist despite the surrounding urban growth (Rand and Rand, 1966). Another positive aspect of *Tropidurus* lizards for studies focusing on phenotypic aspects of species found in urban sites is the extensive literature available for eco-morphology (e.g., Kohlsdorf et al., 2001; Kohlsdorf et al., 2008; Grizante et al., 2010; Tulli et al., 2016), behavior (e.g., Leirião et al., 2019; Rodrigues and Kohlsdorf, 2019), locomotion (Kohlsdorf and Navas, 2012; Brandt et al., 2015) and physiology (Longhini et al., 2019) in this group, including information for development (Py-Daniel et al., 2017; Rossigalli-Costa et al., 2021) and thermal relationships (Kohlsdorf and Navas, 2006; Leirião et al., 2019; Piantoni et al., 2019) of species found in urban areas. In this study, we isolated temperature effects from other environmental parameters likely affected by urbanization (e.g., hydrological regimes or pollutants) and evaluate developmental plasticity in offspring of a *Tropidurus* species commonly found in Brazilian cities. We developed *T. catalanensis* embryos in different thermal regimes in the laboratory [Developmental Environments (DE): cold = 24°C and warm = 30°C], to test the hypothesis that temperature during development affects the offspring phenotypic profile, evaluated from locomotor performance and morphological traits. Results provide evidence for phenotypic effects of developmental environments in morphological features among neonates of *T. catalanensis* incubated in different thermal regimes, with consequences for locomotion. The physiological and morpho-functional implications of different developmental conditions may be decisive for the permanence of *T. catalanensis* lizards in urbanized areas, a discussion that contributes for a better understanding of how native species cope with environmental shifts that might be accentuated in urbanized sites.

## Materials and Methods

We evaluated the effects of developmental environments in the phenotypic profile of neonate lizards focusing on two sets of traits: 1) locomotor performance and 2) morphology. After incubating eggs of *T. catalanensis* in two different thermal regimes [Developmental Environments (DE): cold = 24°C and warm = 30°C], we measured sprint speeds at two orientations (horizontal and vertical surfaces) and three different temperatures [Test Temperatures (TT) = 24°C, 30^o^C and 36°C], assumed as a proxy of essential free-living activities, and quantified body size and limb and tail relative proportions. Data acquisition, experimental design and statistical analyses are described along this section.

### Egg Collection and Incubation

A total of 20 gravid females of *T. catalanensis* were collected by lasso (Van Sluys, 1993) at the Brazilian city of São Simão, in the state of São Paulo (21° 28′ 44″ S 47° 33′ 03″ W), which was founded in 1824 and experienced intensified urbanization in the past two decades. Animals were transported in cloth bags to the Animal Facility of the Laboratory of Integrative Biology and Evolution (University of São Paulo at Ribeirão Preto, SP, Brazil) in the same day they were collected. Females were maintained in a maximum of three individuals per terrarium, which consisted of plastic boxes (40 cm × 40 cm × 60 cm) covered with a 15 cm layer of moist medium-grain vermiculite, until oviposition. Food was provided every two days and water was offered *ad libitum*; thermoregulation at a 12 h dark:light cycle was granted by incandescent UVA/UVB (for vitamin D production) and 60 W incandescent lamps (adapted from Sanger et al., 2008). We checked each terrarium daily for eggs, and carefully examined each female to identify which individual laid a given clutch in order to control for genetic and maternal effects. From the 20 clutches obtained (one from each female), we distributed 80 eggs into small plastic cylindric containers of 145 ml (7.5 cm × 5.0 cm) filled with moist vermiculite. We perforated the lids to enable air renovation and fixed the water potential at -150 cmkPa, replacing eventual evaporated water (based on Steele et al., 2018 and following; Rossigalli-Costa et al., 2021). To avoid genetic bias, we separated the clutches and equally distributed the eggs between two developmental environments: 24°C (cold, N = 40) and 30°C (warm, N = 40); we did not consider the thermal regime of 36°C (overheat) as a third developmental condition because this temperature seems unfeasible for the development of *T. catalanensis* embryos (see Rossigalli-Costa et al., 2021). Due to the lack of information for nest temperatures in *T. catalanensis*, we chose developmental temperatures within the range of air, substrate and body temperatures recorded for *Tropidurus* lizards at São Simão and other Brazilian cities where this species is found (see Kohlsdorf and Navas, 2006; Leirião et al., 2019; Maia-Carneiro and Navas, 2021), which also agree with the range of preferred temperatures recorded for *Tropidurus* lizards in thermal gradients (31°C to 37°C, see Kohlsdorf and Navas, 2006). Incubation temperatures were controlled by Bio-Oxygen Demand (BOD) incubators (Eletrolab^®^), and the precision of thermal regimes was confirmed using data loggers (NOVUS^®^). Eggs were daily inspected for monitoring the development of the embryos, being maintained in the incubators until hatching. These procedures followed Rossigalli-Costa et al. (2021).

### Locomotor Performance

We started locomotor performance tests seven days after hatching. Until performing the locomotion trials, neonates were maintained in terraria at conditions similar to those described for gravid females. Specifically, they were placed in terraria maintained at a 25°C acclimatized room and 60 W incandescent lights were provided in each terrarium to grant thermoregulation. Photoperiod was established at a 12 h dark:light cycle. Tests were performed in an acclimatized room, and we ran each lizard individually. A test comprised one horizontal and one vertical race from each lizard in a given temperature [Test Temperatures (TT) = 24°C, 30°C, and 36°C], in a total of two races in a given day for each individual. Tests were intercalated every two days, and test temperatures were repeated at two different days. At the end of all locomotor performance tests, we gathered two sprint speeds measurements at each temperature (24°C, 30°C, and 36°C) and orientation (horizontal or vertical) for each individual, obtained in different days (total of 12 records for each lizard). The Test Temperatures chosen reflect the thermal variation experienced by *Tropidurus* lizards during daily hours of activity (Maia-Carneiro and Navas, 2021). We randomized the order of tests (i.e., in which temperature lizards ran) and also the orientation (horizontal or vertical) they started running each day. Before each race, lizards were maintained for at least one hour at the testing temperature until reaching body temperatures near those of the test (confirmed using an infrared thermometer). We recorded sprint speeds at horizontal and vertical directions of 20 neonates - ten from ‘cold DE’ (24°C) and ten from ‘‘warm DE’’ (30°C). From the total of 80 eggs incubated, 36 neonates from 30°C and 34 neonates from the cold developmental environment of 24°C actually hatched. We ran all the 70 neonates that hatched, but several individuals refused to run at one or more trials at a given temperature, and the time window we considered one lizard a ‘‘neonate’’ was 15 days. Therefore, and in order to include only individuals that performed satisfactorily at the three testing temperatures to infer running thermal sensitivity, our final dataset comprised races obtained for 10 lizards from each developmental environment. The horizontal sprint speed was measured at a pine board racetrack (90 cm length × 25 cm width × 35 cm high). The camera was placed 2 m above the center of the racetrack and captured its entire length. Vertical sprint speed was performed at 80 cm high × 25 cm width concrete blocks leaning on the wall, with the camera placed at a 2 m frontal distance from the blocks. For both tests, neonates were placed on the track and stimulated by to run by hand if they did not flee immediately; sprints speeds were recorded until lizards reached the end of the track. All races were recorded with a high-speed digital camera at 120fps (Canon^®^ Sx50hs). Videos were analyzed using the Tracker^®^ video analyzing software, in which we extracted the total time to complete the races (measured in seconds), maximum and average sprint speed (m/s) and maximum and average acceleration (m/s^2^) from each trial.

### Morphological Measurements

We compiled a dataset of morphological measurements obtained in each lizard immediately after hatching to evaluate the effects of developmental conditions on body size and associated proportions. We avoided measuring animals along the days of performance tests to minimize stress from excessive manipulation. All data in this study were obtained in the first two weeks after hatching, and during this time window neonate lizards barely feed and seem to rely on remaining embryonic energetic reserves; in the lab, growth of *T. catalanensis* during the first days after hatching rarely exceeds 5% of their initial size. We focused on traits related to locomotor performance in *Tropidurus* lizards (see Kohlsdorf et al., 2001; Grizante et al., 2010; Kohlsdorf and Navas, 2012): Hand and Feet lengths (from the base of the wrist/ankle to the distal phalanx of Digit IV); Fore and Hindlimb lengths (the sum of humerus and radius/femur and tibia lengths); Height and Width of the Pectoral and Pelvic Girdles; Trunk length; Tail length and Snout-Vent length (SVL, a proxy of overall body size). Traits were measured externally and congregate osteological, muscular, and epithelial elements; all measurements were performed by the same researcher using a digital calliper (Mitutoyo Inc. ± 0.01 mm).

### Statistical Analyses

Analyses were performed based on two parameters of sprint performance: 1) maximum sprint speed (Vmax; the maximum sprint speed the individual reached in each testing temperature); and 2) absolute maximum sprint speed (Absolute Vmax, the highest sprint speed value reached amongst all test temperatures). We also analyzed our data separately considering the thermal sensitivity of locomotor performance and functional associations with morphological traits, as further described. All analyses were performed using RStudio (1.4.1717) in R (version 4.0.5 - R Core Team, 2021).

We first performed descriptive analyses of the total duration of the race (in seconds), Vmax (m/s) at each test temperature and Absolute Vmax (m/s). A generalized linear mixed model (glmm; package *glmmTMB*, Brooks et al., 2017) was used to evaluate relationships of Vmax with surface orientation (vertical or horizontal), developmental environment (DE: cold or warm), and testing temperature (TT: 24°C, 30°C, and 36°C). Interactions between orientation, developmental environment (DE) and testing temperatures (TT) were considered in the models, and ‘‘individual’’ and ‘‘clutch’’ (maternal effects) were considered as random intercepts. When significant interaction effects were detected, we performed post-hoc pairwise analyses using the *emmeans* function (Russell, 2021). We corrected *p*-values for multiple comparisons using the Tukey method to compare a family of three estimates. Prior to these steps, we selected the statistical family distribution that best fitted our model through the Akaike information criterion and proceeded with the analyses using the gamma distribution. To visualize the thermal sensitivity of locomotor performance, we performed a linear regression, using *ggplot2* (Wickham, 2016), between Vmax (m/s) at horizontal and vertical orientation and the body temperature (in °C) the animals reached during the locomotion trials (after being maintained for 1 h at the test temperature).

The morphological database was evaluated using a multivariate approach. First we extracted size-adjusted residuals of shape (see Shingleton, 2010), and then we performed a Principal Component Analysis (PCA) using the package *vegan* (Oksanen et al., 2019). We selected the Principal Components (PCs) having eigenvalues higher than 1 (PCs 1–4, which together explained 78.01% of the total variation) and plotted the corresponding scores with the predictor variable ‘‘development environment’’ (DE) for visual inspection of the morphospace (packages *ggplot2* from Wickham, 2016 and *ggord* from Beck, 2021). We tested for morphological differences between individuals incubated at different developmental environments performing an analysis of variance (ANOVA) on the PC scores.

Associations between the morphological profile of neonates and locomotor performance were tested using linear models with the PCs scores as a function of Absolute Vmax, after selecting the best models for our data. In the models, Absolute Vmax was included as the response variable and different combinations of the four morphological PCs, the developmental environment (DE), and surface orientation (vertical or horizontal) were considered as predictor variables. We applied an automated model selection for each complex model using the package *glmulti* (Calcagno, 2020), starting from [glm (Absolute Vmax ∼ PC1 + PC2 + PC3 + PC4 + DE + Orientation)], and then sequentially removing variables that explained the least variation in each model tested (see Barros et al., 2021 for an example of similar analyses). The best models and their AICc support were used for model selection. For all models, ‘‘clutch’’ and ‘‘individual’’ were fixed as random effects. Integrated effects of the predictor variables on Absolute Vmax were tested using *glmm*. All analyses using PCs were size-corrected, so we complemented our statistical design using ANCOVAs to test Absolute Vmax (horizontal and vertical separately) as a function of integrated effects of body size (SVL) and DE, in order to address morpho-functional implications from the effect of DE on growth. All morphology-related models were tested using the Akaike Criterion Information for the best statistical family distribution and were implemented in the *glmmTMB* package (Brooks et al., 2017). Under request, the authors will provide detailed information on analyses and codes as an Rmarkdown file.

## Results

### Locomotor Performance of *Tropidurus* Neonates From Different Developmental Environments: Relationships With Test Temperatures

**FIGURE 1:**
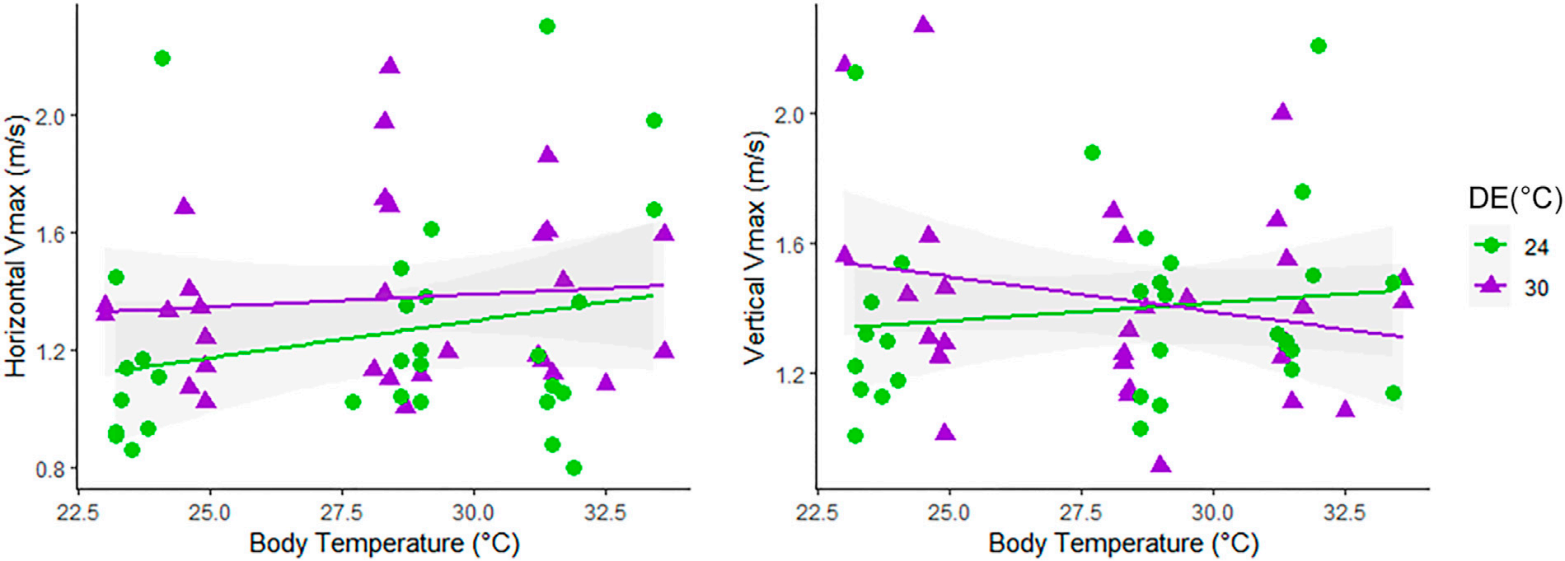
Thermal performance of maximum sprint speeds (Vmax) in lizards running at Horizontal and Vertical orientations in the body temperature reached at the Testing Temperature (TT) of 24°C, 30°C, and 36°C. Animals from different developmental environments (DE) coded as follows: 24°C, green dots; 30°C, purple triangles.

Races performed at the vertical wall (80 cm length) overall lasted longer than those at horizontal orientation (90 cm length), as detailed in Table 1 and Supplementary Table S1. In the locomotor trials, neonates reached body temperatures near 23.81 ± 0.62 (TT = 24°C), 28.38 ± 0.86 (TT = 30°C), and 31.80 ± 0.88 (TT = 36°C). We first evaluated thermal sensibility of locomotion by plotting Vmax at each orientation as a function of the body temperature reached at locomotor performance trials (Figure 1). Visual inspection of the curves suggested slight variation in the thermal sensitivity of Vmax between *Tropidurus* lizards from distinct developmental environments in both orientations (horizontal and vertical), despite the considerable variation among individuals observed in the graphs. When effects of Developmental Environment (DE), Test Temperature (TT), or the interaction among factors (Orientation*DE*TT) on maximum sprint speeds were tested separately, we did not identify any significant association (Supplementary Table S2). Optimal temperature for sprint speed was not consistent among individuals, even within the same developmental condition (Figure 1), so we used the highest value of maximum speed from all tests (Absolute Vmax) to evaluate variation in locomotor performance and morpho-functional relationships between neonates of *Tropidurus* lizards incubated at different thermal regimes.

**TABLE 1:** Locomotor performance (mean and standard error) of *Tropidurus catalanensis* neonates raised in different developmental environments (DE).

	TT	Horizontal		Vertical	
		Total time of running (s)	Vmax (m/s)	Total time of running (s)	Vmax (m/s)
DE 24°C	24°C	2.77 ± 0.23	1.17 ± 0.19	5.33 ± 0.41	1.34 ± 0.17
	30°C	3.09 ± 0.30	1.24 ± 0.14	5.69 ± 0.68	1.39 ± 0.16
	36°C	2.85 ± 0.27	1.33 ± 0.22	5.28 ± 0.54	1.44 ± 0.17
De 30°C	24°C	2.51 ± 0.23	1.28 ± 0.13	4.74 ± 0.49	1.53 ± 0.19
	30°C	3.01 ± 0.36	1.44 ± 0.20	4.43 ± 0.44	1.31 ± 0.15
	36°C	3.12 ± 0.37	1.21 ± 0.16	5.19 ± 0.32	1.36 ± 0.16

Total time of running and maximum sprint speed (Vmax) values separated according to the two temperatures embryos were developed (DE) and the three test temperatures (TT) in which the locomotor tests were performed.

### Morphological Profiles and Functional Relationships in Neonates From Different Developmental Environments

**FIGURE 2:**
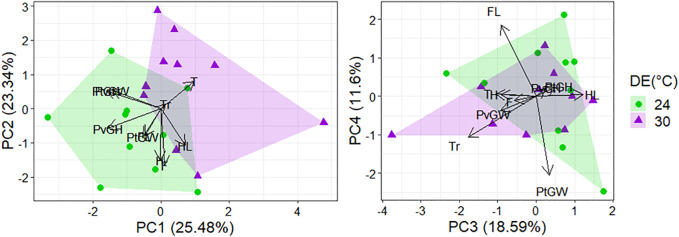
Principal component analysis of morphological traits measured in 20 juveniles from the warm DE (30°C; purple triangles) and the cold DE (24°C; green dots). Components with eigenvalues higher than one were retained, and loadings of variables strongly associated to each PC are detailed in Table 2. The scores of PC2 and PC3 differed between neonates from the cold and warm DE (PC2: *p* = 0.001; PC3: *p* = 0.004). Codes for morphological traits are: H, Hand length; F, Foot length; FL, Frontlimb length; HL, Hindlimb length; PtGH, Pectoral Girdle Height; PtGW, Pectoral Girdle Width; PvGH, Pelvic Girdle Height; PvGW, Pelvic Girdle Width; Tr, Trunk length; T, Tail length.

The morphological profiles of *T. catalanensis* neonates differed according to the thermal regime eggs were incubated (raw data presented at Supplementary Table S3). Lizards from the warm Developmental Environment (DE = 30°C) were bigger (i.e., larger SVL) than those from the cold DE of 24°C (*p* < 0.0001). The Principal Components Analyses performed with body proportions (size-adjusted residuals) retrieved four Principal Components (PCs) with eigenvalues higher than 1.0, which, together, explained 78.01% of total variation. The two first principal components explained together almost 50% of the variation (Table 2) and were mostly associated to variation in pelvic and pectoral girdles (PC1) and limbs (foot, hand and hindlimb, PC2). Among the four PCs retained, PC2 (hand, foot and hindlimb lengths) and PC3 (hindlimb, trunk and tail lengths) differed between individuals from distinct DEs (PC1: *p* = 0.381; PC2: *p* < 0.0001; PC3: *p* = 0.004, PC4: *p* = 0.960; see also Figure 2).

**TABLE 2:** Principal component analysis indicating the percentage of explained variance of each component retained.

Loadings	PC1	PC2	PC3	PC4
Hand	−0.009	−**0.502**	−0.305	0.015
Frontlimb Length	−0.157	−0.263	−0.305	**0.617**
Foot	0.034	−**0.552**	−0.190	−0.038
Hindlimb Length	0.235	−**0.356**	**0.408**	0.012
PectG. Height	−**0.536**	0.174	0.164	0.075
PelvG. Height	−**0.519**	−0.192	0.074	0.065
PectG. Width	−0.174	−0.274	0.114	−**0.682**
PelvG. Width	−**0.475**	0.180	−0.308	−0.126
Trunk	0.045	0.046	−**0.589**	−**0.355**
Tail	0.324	0.266	−**0.352**	0.018
Eigenvalues	1.596	1.528	1.364	1.077
Proportion of var. (%)	25.48%	23.34%	18.59%	11.60%

Only components with eigenvalues higher than one were considered. Values in bold indicate traits having higher loads associated to a given PC.

**FIGURE 3:**
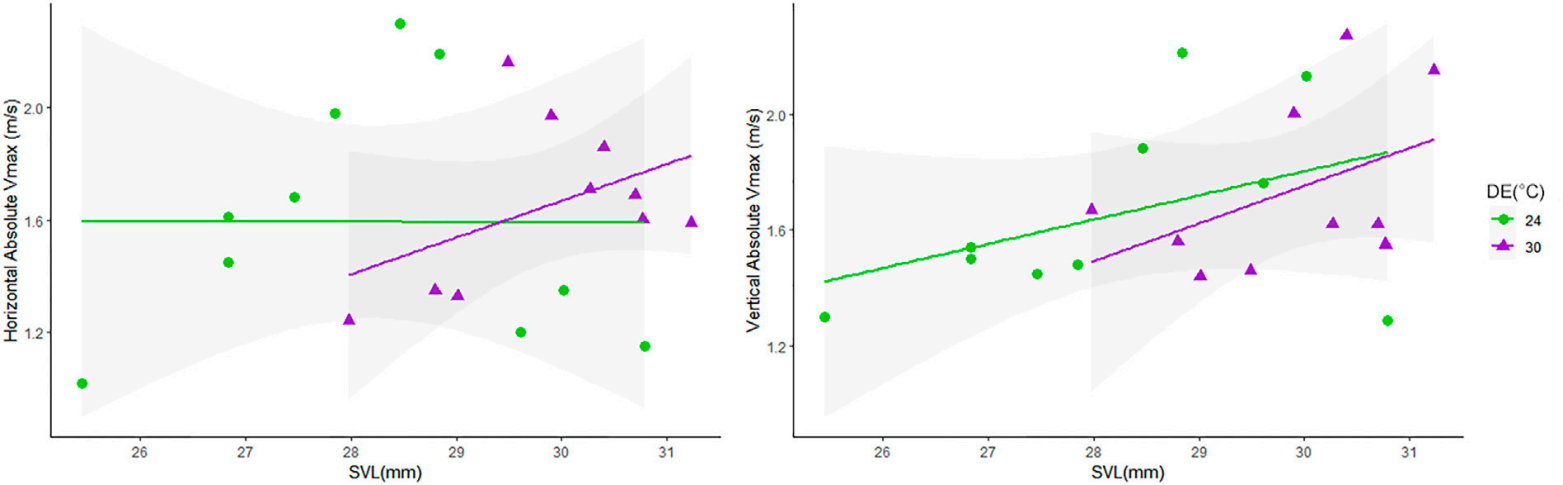
Values of Absolute Vmax (m/s) in relation to body size (SVL) at two orientations (Horizontal and Vertical). Lizards from the warm DE (30°C, represented by purple triangles) had larger SVL than those from the cold DE (represented by green dots), and body size positively affected Absolute Vmax at vertical races (*p* = 0.0225).

Relationships between locomotor performance (Absolute Vmax) and body size (also considering effects of DE on SVL) were tested using ANCOVA, as lizards from the warm DE were larger (see Figure 3 and Supplementary Figure S1). We identified effects of body size on Absolute Vmax at vertical orientation (*p* = 0.0225), but no effects were detected from the interaction between SVL and DE (*p* > 0.05) in the speeds recorded at this orientation; when running at horizontal orientation, no effects on Absolute Vmax were detected from body size or its interaction with DE (Figure 3).

Functional relationships of locomotor performance in neonates from different developmental environments were better explained by the model comprising two morphological components (PC3 and PC4) and the Developmental Environment (DE) (Model 1: Absolute Vmax∼1 + PC3+PC4+DE; AICc = 9.647, weight = 0.161; Table 3). Morphological traits with higher loads in PC3 (18.59% of total variation) were hindlimb trunk and tail lengths, and those in PC4 (11.6% of total variation) were frontlimb and trunk lengths and pectoral girdle width (Table 2). We tested for specific associations between Absolute Vmax and the three factors of the best model selected (M1) - PC3, PC4, and DE - and all were statistically significant (Table 3). To summarize, our results provide evidence for associations between Absolute Vmax and body size (SVL) and relative proportions (limbs, trunk, and tail lengths, and pelvic girdle width) that are associated with the thermal environment where lizards developed, suggesting that effects of environmental conditions at early developmental stages on morphological traits also impact locomotor performance.

**TABLE 3:** Results of the three best generalized mixed models selected for morpho-functional relationships of Absolute Vmax.

Model		AICc	Weight	Factor	Chisq	Df	Pr (>Chisq)
M1	**AbsVmax∼1 + PC3 + PC4 + DE**	9.647	0.161	PC3	6.568	1	**0.010**
				PC4	40.996	1	**0.000**
				DE	4.758	1	**0.029**
M2	AbsVmax ∼1 + PC2 + PC3 + PC4 + DE	10.179	0.123				
M3	AbsVmax ∼1 + PC3 + PC4	10.713	0.094				

The best-fit model (M1, highlighted) grouped two morphological components (PC3 [hindlimb, trunk and tail lengths] and PC4 [frontlimb and trunk lengths and pelvic girdle width) and DE. Values in bold correspond to the significance value (*p* < 0.05) between Absolute Vmax and each predictor variable. All predictor variables influenced the Absolute Vmax.

## Discussion

Thermal regimes are particularly important for embryos developing in environments susceptible to external conditions, including oviparous lizard species, because temperature may affect the establishment of features directly related to fitness. Environmental changes in specific settings due to urbanization processes received increasing attention in the past decades, due to expected impacts on biodiversity derived from differences in rates of sun exposure, shade availability, and structural components of the habitat during city growth (e.g., McDonnell and Hahs, 2015; Winchell et al., 2017; Rivkin et al., 2019; Miles et al., 2021), as well as variation in biotic interactions that include predation and structure of the community (Birnie-Gauvin et al., 2016; Andrade et al., 2018). Although urbanization processes may negatively affect native species and consequently demand conservation actions (see Bradley and Altizer, 2007; McKinney, 2008), some species benefit from new environmental contexts that represent increments in the availability of ecological niches (Eriksson, 2013).

In the present study, we evaluated effects of developmental temperatures in the phenotypic profiles of a lizard commonly found in Brazilian cities, inferring functional implications for locomotor performance in neonates of *Tropidurus catalanensis* incubated in different thermal regimes. Our results provide evidence that the thermal regime experienced during development influences the morphological profile of *T. catalanensis* neonates, which indirectly affects locomotor performance. Specifically, individuals from warm conditions were larger, and body size affected absolute maximum speeds of *T. catalanensis* especially at vertical surfaces. Moreover, morphological traits associated to PC2 (hand, foot and hindlimb lengths) and PC3 (hindlimb, trunk and tail lengths) that differed between individuals incubated at different temperatures correspond to variables often interpreted as adaptations to urbanization in other lizard species (see Winchell et al., 2016; Winchell et al., 2018b). Although the *Tropidurus* lizards overall ran at similar speeds in horizontal and vertical races and analyses performed on isolated traits did not indicate significant interactions of locomotor performance with Developmental Environment or Test Temperatures, neonates from the warm DE (30°C) were larger, and body size affected Absolute Vmax at vertical orientation. Moreover, some of the morphological traits that differed between neonates from different DEs (PC3: hindlimb, trunk and tail lengths) were associated with the best-fit model for morpho-functional associations of Absolute Vmax. Interestingly, effects of the DE on locomotor performance were only detected when the morphological profile of neonates was considered in the analyses, regardless of the test temperature. We performed locomotor trials at 24°C, 30°C, and 36°C, and most lizard species select preferred temperatures between 25°C and 35°C and exhibit sprint speeds usually optimized between 32°C and 36°C (Huey and Bennett, 1987). Lizards of *T. catalanensis* are diurnal and heliophilic, and experience temperatures between 25°C and 36°C during daily activities that include thermoregulation, foraging, and reproduction (Maia-Carneiro and Navas, 2021). Despite the lack of information about thermal regimes in the nests of *T. catalanensis*, literature records indicate an average temperature near 29°C in nests of other lizards found in urban areas, such as *Anolis cristatellus* (Tiatragul et al., 2020). Although the two developmental thermal regimes used here are within the range of field temperatures recorded for *Tropidurus* lizards (Kohlsdorf and Navas, 2006; Leirião et al., 2019; Maia-Carneiro and Navas, 2021), the developmental temperature of 24°C established in the lab for *T. catalanensis* might be interpreted as a cold environment, especially considering that preferred temperatures for tropidurine lizards are usually above 30°C (Kohlsdorf and Navas, 2006).

Morphological differences in Tropidurinae lizards are often associated to ecological divergence (Kohlsdorf et al., 2001; Grizante et al., 2010) and explain interspecific variation in locomotor performance (Kohlsdorf and Navas, 2012). In this study, most of the morphological variation among *Tropidurus* neonates from different developmental environments concentrates in two principal components that aggregate height and width of pelvic and pectoral girdles (PC1) and limb, hand and feet relative proportions (PC2). Locomotor performance was indirectly affected by the effects of incubation temperature in two other Principal Components (PC3 and PC4), which aggregate limb trunk and tail lengths and the pectoral girdle width. Interestingly, these parameters correspond to morphological traits interpreted in *Anolis* lizards as adaptive to urbanization (trunk width, limb lengths and autopodial features; see Winchell et al., 2016; Winchell et al., 2018). Our results therefore foster future studies addressing the role of developmental plasticity in adaptive processes of *T. catalanensis* populations in Brazilian urbanized sites.

Morphological and physiological changes mediate evolutionary processes in natural populations that may lead to local adaptation and rapid evolution in response to environmental changes (Winchell et al., 2016; Winchell et al., 2018; Noble et al., 2021). Despite expected negative effects of urbanization for native species (see McDonnell and Hahs, 2015), high densities of *T. catalanensis* lizards are observed in several Brazilian cities. Part of the success to remain in these areas despite rapid urbanization process may reside on how animals cope with the effects of developmental temperature on the overall morphological profile of neonates and their consequences for morpho-functional associations of sprint performance*.* We recognize that isolated effects of developmental environments in these lizards may influence other features related to fitness at different ages, but individual survival in the critical ontogenetic window immediately after birth might be granted by stability of sprint performance morpho-functional associations that likely enhances chances of escaping from predators (e.g., cats and birds) in Brazilian cities. Warmer developmental environments produce larger *Tropidurus* neonates, which might be favored in urban environments also due to the positive association between sprint speeds and body size. Studies evaluating how native species successfully remain in anthropized fields perfectly complement data available for the negative effects of urbanization that might lead to local extinction of specific lineages (see Winchell et al., 2018; Sparkman et al., 2018; Putman et al., 2019; Vaughn et al., 2021 for recent discussions). Our findings highlight the importance of a new dimension to this discussion (i.e., thermal effects in developmental processes), providing evidence that rapid shifts in specific environmental parameters, such as temperature, may influence the development of isolated traits in *T. catalanensis* and consequently affect morpho-functional relationships in the species, an aspect that must be considered by studies addressing how these lizards cope with habitats that change fast, as those suffering rapid urbanization due to city growth.

## Data Availability

The original contributions presented in the study are included in the article/Supplementary Material, further inquiries can be directed to the corresponding author.
