# Supplementary material for: Native Lizards Living in Brazilian Cities: Effects of Developmental Environments on Thermal Sensitivity and Morpho-Functional Associations of Locomotion
Source: Front Physiol. 2022 Jul 15;13:891545. doi: 10.3389/fphys.2022.891545 (PMC9335278; doi:10.3389/fphys.2022.891545)
Supplement: Supplementary file 1 [file DataSheet1.pdf]

Supplementary Tables

Supplementary Table 1. Mean and standard error of locomotor performance data at each experimental condition separated by the temperature that embryos developed. Data refers to the time (in seconds) to complete the 90cm (horizontal running) or 80cm (vertical running) of the running track.

	**Developed at 24°C**			**Developed at 30°C**		
	**24°C**	**30°C**	**36°C**	**24°C**	**30°C**	**36°C**
**Horizontal**						
Total time of the race (s)	2.77±0.23	3.09±0.30	2.85±0.27	2.51±0.23	3.01±0.36	3.12±0.37
Max Sprint Speed (m/s)	1.17±0.19	1.24±0.14	1.33±0.22	1.28±0.13	1.44±0.20	1.21±0.16
Mean Sprint Speed (m/s)	0.31±0.07	0.29±0.09	0.30±0.08	0.33±0.09	0.35±0.12	0.29±0.11
Max Sprint Acceleration (m/s^2)	86.89±3.05	71.41±2.40	66.69±1.99	57.96±1.48	72.88±1.88	67.12±3.01
Mean Sprint Acceleration (m/s^2)	5.47±0.41	6.35±0.43	5.85±0.48	7.34±0.44	7.16±0.39	6.35±0.55
**Vertical**						
Total time of the race (s)	5.33±0.41	5.69±0.68	5.28±0.54	4.74±0.49	4.43±0.44	5.19±0.32
Max Sprint Speed (m/s)	1.34±0.17	1.39±0.16	1.44±0.17	1.53±0.19	1.31±0.15	1.36±0.16
Mean Sprint Speed (m/s)	0.17±0.06	0.22±0.10	0.20±0.09	0.21±0.08	0.23±0.10	0.24±0.07
Max Sprint Acceleration (m/s^2)	89.54±2.43	79.11±1.46	75.38±1.30	97.56±2.33	92.52±1.99	76.06±1.25
Mean Sprint Acceleration (m/s^2)	8.62±0.44	9.42±0.55	9.26±0.55	9.44±0.43	8.98±0.64	9.78±0.36

Supplementary Table 2. Results of generalized mixed models evaluating sprint speeds (Vmax) as a function of running orientation, developmental environment (DE), test temperature (TT) and also considering interactions among factors.

	Chisq	Df	Pr(>Chisq)
Orientation	4.044	1	0.04434
DE	1.117	1	0.29052
TT	1.095	2	0.57850
Orientation:DE	1.033	1	0.30948
Orientation:TT	2.528	2	0.28259
DE:TT	1.333	2	0.51351
Orientation:DE:TT	1.825	2	0.40154

Supplementary Table 3. Morphological traits (mean and standard error) in neonates of *T. catalanensis* from different developmental environments (DE=30°C and DE=24°C). Codes: PectG=Pectoral Girdle; PelvG=Pelvic Girdle.

	**30°C**	**24°C**
**Hand length**	6.49 ± 0.21	6.69 ± 0.17
**Frontlimb length**	10.08 ± 0.25	10.62 ± 0.27
**Foot length**	10.09 ± 0.25	10.30 ± 0.21
**Hindlimb length**	12.95 ± 0.24	12.75 ± 0.30
**PectG.Heigh**	4.34 ± 0.20	4.86 ± 0.22
**PelvG.Heigh**	3.55 ± 0.21	4.12 ± 0.20
**PectG.Width**	5.45 ± 0.19	5.80 ± 0.22
**PelvG.Width**	4.53 ± 0.20	4.58 ± 0.17
**Trunk length**	13.12 ± 0.34	12.40 ± 0.25
**Tail length**	44.88 ± 0.45	39.58 ± 0.54
**SVL**	29.86 ± 0.32	28.21 ± 0.41

Supplementary Figures

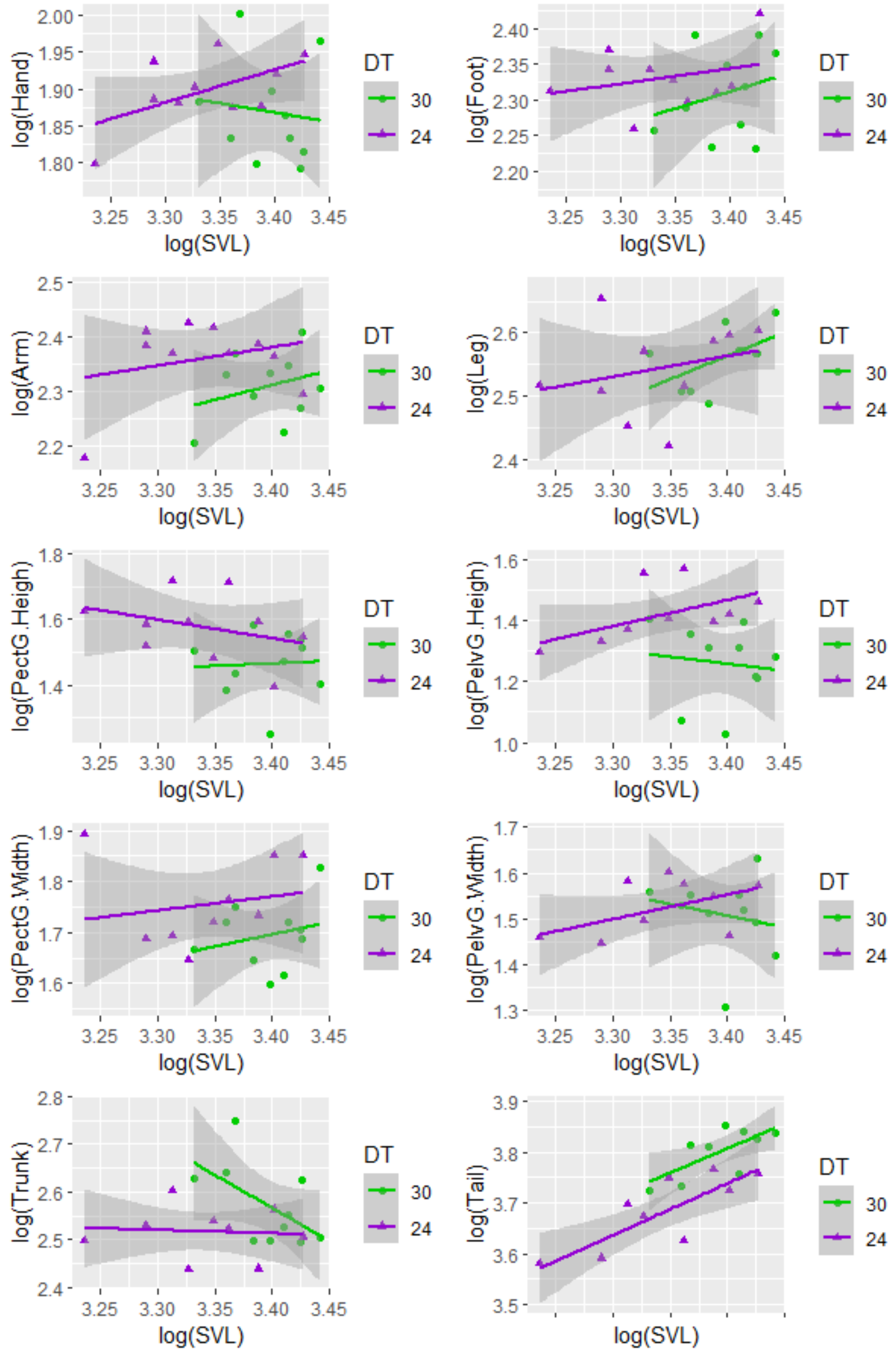

Supplementary Figure 1. Allometric relationships between morphological traits and SVL (log transformed) in neonates raised at two different development temperatures (DT, in °C). Abbreviations correspond to: PectG = Pectoral Girdle; PelvG = Pelvic Girdle.
